# Pernicious Anemia Presenting as Pseudo-Thrombotic Microangiopathy: A Case Report

**DOI:** 10.7759/cureus.110006

**Published:** 2026-05-31

**Authors:** Sai Sushrutha Mudupula Vemula, Shreeya Adhikari, Shreya Motkur, Alfarooq Alshaikhli, Borys Hrinczenko

**Affiliations:** 1 Internal Medicine, Michigan State University, East Lansing, USA; 2 Internal Medicine, Sparrow Hospital, Lansing, USA; 3 Internal Medicine, University of Texas Rio Grande Valley, Edinburg, USA; 4 Hematology and Medical Oncology, McLaren Greater Lansing, Lansing, USA; 5 Oncology, Michigan State University, East Lansing, USA

**Keywords:** acute hemolytic anemia, hemolytic anemia, pernicious-anemia, pseudothrombotic microangiopathy, pseudo-ttp, vit b12 deficiency

## Abstract

Thrombotic thrombocytopenic purpura (TTP) is a rare, life-threatening thrombotic microangiopathy (TMA) characterized by microangiopathic hemolytic anemia, thrombocytopenia, and end-organ dysfunction, with high untreated mortality that can be effectively reduced with timely plasma exchange (PLEX) and immunosuppressive therapy. However, several conditions can produce a clinically similar pseudo-TMA picture, among which severe vitamin B12 deficiency is an underrecognized and potentially treatable mimic that occurs due to ineffective intramedullary erythropoiesis, megaloblastic fragmentation of structurally abnormal erythrocytes, and pancytopenia in the absence of true endothelial injury or ADAMTS13 deficiency. We present a 74-year-old female patient who presented with altered mental status, bradycardia, and severe hemolytic anemia, in whom PLEX was initiated for suspected TTP but failed to improve platelet counts despite four sessions, ultimately prompting a workup that showed B12 deficiency due to pernicious anemia, confirmed by an elevated methylmalonic acid (MMA) and low serum B12, with complete hematologic recovery following cyanocobalamin repletion.

## Introduction

Vitamin B12 is a water-soluble vitamin essential for DNA replication, myelin synthesis, and cell division, making rapidly proliferating tissues, such as hematopoietic cells, particularly vulnerable to deficiency. Deficiency is formally defined as serum levels below 200 pg/mL; however, functional deficiency may occur at levels below 300 pg/mL in symptomatic individuals with elevated methylmalonic acid (MMA) and homocysteine [1]. The Framingham Offspring Study notably found that nearly 40% of adults had low-normal B12 levels [2]. Pernicious anemia, an autoimmune condition caused by destruction of gastric parietal cells leading to intrinsic factor deficiency and impaired ileal cobalamin absorption, accounts for 20-50% of documented vitamin B12 deficiency in adults, with a general population prevalence of 0.1%, rising to 1.9% in adults over 60, and a predilection for females and individuals of Northern European ancestry [3-6]. While classically presenting with megaloblastic anemia and neurologic manifestations, severe B12 deficiency can rarely produce profound hematologic abnormalities, including hemolytic anemia, thrombocytopenia, and peripheral schistocytosis from ineffective erythropoiesis mimicking thrombotic thrombocytopenic purpura (TTP) [7].

TTP is a rare, life-threatening thrombotic microangiopathy (TMA) with an incidence of 1-13 cases per million, predominantly affecting women (2:1), adults over 40, individuals of African American descent, and pregnant individuals, with congenital forms rarely presenting in childhood [8,9]. It is classically characterized by the clinical pentad of fever, thrombocytopenia, microangiopathic hemolytic anemia, renal dysfunction, and neurological impairment due to a congenital or acquired deficiency of ADAMTS13, the von Willebrand factor (VWF)-cleaving protease that processes ultralarge VWF multimers released from endothelial cells. In the absence of adequate ADAMTS13 activity, these large multimers accumulate in the circulation, bind to platelet glycoprotein receptors, cause spontaneous platelet aggregation, widespread microvascular thrombosis, and consequent end-organ ischemia and damage [10]. Untreated mortality exceeds 90% but is reduced to ~10-15% with timely plasma exchange (PLEX) and immunosuppressive therapy; accordingly, guidelines recommend immediate PLEX and ICU-level care upon clinical suspicion, without awaiting confirmatory ADAMTS13 testing [11,12].

Fewer than 100 cases have been reported since the initial description, with pseudo-TMA estimated to account for ~2-3% of patients initially suspected to have TTP [12]. Misclassification exposes patients to unnecessary interventions like central venous catheter placement, PLEX, blood product transfusion, ICU admission, while delaying definitive B12 replacement therapy and prolonging hospitalization. This case highlights a critical diagnostic pitfall in a patient presenting with MAHA and thrombocytopenia, underscoring the need for a thorough evaluation strategy to rapidly differentiate pseudo-TMA from true TTP.

## Case presentation

A 74-year-old female patient with a past medical history significant for hypertension, hyperlipidemia, diabetes mellitus, anxiety, and hypothyroidism presented with altered mental status and unresponsiveness. During emergency medical services transport, she was noted to be bradycardic and hypotensive, requiring vasopressor support and transcutaneous pacing. Upon arrival at the emergency department, she was intubated for airway protection.

Initial laboratory evaluation revealed severe pancytopenia, including severe macrocytic anemia (hemoglobin 2.7 g/dL, mean corpuscular volume 138 fL), leukopenia (white blood cell count 2.8 ×10⁹/L), and thrombocytopenia (platelet count 19,000/µL). Other pertinent labs included the following: lactate, 10.8 mmol/L (reference: 0.2-1.8 mmol/L); lactate dehydrogenase (LDH) 1224 U/L (reference: 120-240 U/L); fibrinogen, 203 mg/dL (reference: 150-450 mg/dL); reticulocyte count, 2.1% (reference: 0.63-1.39%); and total bilirubin, 1.7 mg/dL (reference: 0.2-1.2 mg/dL). ABG showed pH, 7.06; pCO2, 35; pO2, 270; sodium, 139 mmol/L (reference: 136-146 mmol/L); bicarbonate, 11 mmol/L (reference: 20.0-31.0 mmol/L); folate, 5.12ng/mL (reference: 3.40-24 ng/mL); and creatinine 2.74 mg/dL (reference: 0.50-1.00 mg/dL; baseline 0.7 mg/dL). Coagulation studies revealed a prothrombin time of 24.0 seconds (reference: 9.5-12.1 s); interquartile range (INR), 2.5 (reference: 2-3); activated partial thromboplastin time (aPTT) of 30.4 seconds (reference: 21.0-31.0 s); and D-dimer, 28.21 mg FEU/L (reference: 0.00-0.74 mg FEU/L), with fibrinogen within normal limits at 203 mg/dL, making overt disseminated intravascular coagulation (DIC) less likely. The patient was resuscitated with five units of packed red blood cells and five units of fresh frozen plasma. Initial imaging, including brain CT and chest, abdomen, and pelvis CT, demonstrated no acute processes. Patient was started on broad-spectrum antibiotics, sepsis workup was sent, and home levothyroxine dose was increased to IV 100 mcg due to suboptimal TSH with normal T3, and T4 in the setting of severe bradycardia.

Hematology was consulted, and further evaluation of her anemia demonstrated evidence of intravascular hemolysis, including elevated lactate dehydrogenase, decreased haptoglobin (<6 mg/dL), and schistocytes on peripheral smear; notably, hypersegmented neutrophils and macro-ovalocytes, classic morphologic hallmarks of megaloblastic anemia, were absent, representing an atypical presentation of severe B12 deficiency. Her PLASMIC score was calculated at 5/7, indicating intermediate risk for TTP. Given clinical concern for TTP, plasmapheresis was initiated, and she was also started on intravenous Solu-Cortef 50 mg every six hours for hemolytic anemia. Blood cultures grew methicillin-resistant *Staphylococcus aureus*, and urine cultures grew *Enterococcus faecalis*, and antibiotics were switched to cefepime and vancomycin. However, platelet count continued to decline despite plasmapheresis for four days, and the patient needed platelet transfusions. ADAMTS13 activity was 41.3%, which was not consistent with severe deficiency; therefore, TTP was excluded. Echocardiogram (ECHO) was negative for any vegetations/infective endocarditis. Vitamin B12 was low at 163 pg/mL (reference: 211-911 pg/mL) on admission; MMA, obtained six days later, was elevated at 985 nmol/L, confirming functional B12 deficiency at the tissue level. Workup for pernicious anemia revealed negative antiparietal cell antibody but elevated anti-intrinsic factor antibody at 123.8 AU/mL, consistent with pernicious anemia causing cobalamin deficiency. She was initiated on cyanocobalamin 1000 mcg daily for seven days, followed by weekly administration for four weeks, and then monthly maintenance therapy, along with tapering of steroids, which improved her hemoglobin to 10.9 g/dL and platelets up to 211 × 109/L, as mentioned in Table 1. The hospital course was complicated by acute kidney injury and hypernatremia, which were treated with free water flushes and IVF, and sacral wounds with no signs of active infection, managed with wound care. With appropriate treatment, her mental status improved, and she was ultimately discharged to a rehabilitation facility with close outpatient hematology follow-up to monitor recovery of her blood counts.

**Table 1:** Key laboratory values before and after vitamin B12 replacement MCV: mean corpuscular volume; LDH: lactate dehydrogenase; WBC: white blood cell

Parameter	Pretreatment	Posttreatment	Reference range
Hemoglobin (g/dL)	2.7	10.9	12.0-16.0
Platelets (×10⁹/L)	19	211	140-400
MCV (fL)	138	104	80.0-100.0
WBC (×10⁹/L)	2.8	7.79	4.0-10.8
LDH (U/L)	1224	462	120-240
Creatinine (mg/dL)	2.74	0.64	0.50-1.00

The diagnosis was established by the combination of severely low vitamin B12 at 163 pg/mL, elevated MMA at 985 nmol/L confirming functional deficiency, absence of ADAMTS13 deficiency, and hematologic recovery following vitamin B12 replacement, collectively distinguishing pseudo-TMA from true TTP.

## Discussion

Pernicious anemia results from autoimmune destruction of gastric parietal cells, leading to loss of intrinsic factor secretion and failure of the intrinsic factor-cobalamin complex to bind cubilin receptors in the terminal ileum, impairing enteral cobalamin absorption [3]. Antiparietal cell antibodies are present in up to 90% of cases but are nonspecific and are seen in other autoimmune conditions like autoimmune gastritis, Hashimoto's thyroiditis, Graves' disease, type 1 diabetes mellitus, and even in some healthy older adults [13]. Anti-intrinsic factor antibodies, though detected in only 60% of patients, carry a specificity exceeding 99% and are diagnostic when positive [13]. In our patient, antiparietal cell antibody was negative, while anti-intrinsic factor antibody was markedly elevated at 123.8 AU/mL, establishing the etiology as pernicious anemia [13,14]. The slow depletion of hepatic B12 reserves over the years explains the severe functional deficiency, confirmed by an MMA of 985 nmol/L, despite a serum B12 that underestimated tissue levels. As the absorptive defect is irreversible, lifelong cobalamin supplementation is required, as seen in our patient who was started on a monthly maintenance cyanocobalamin regimen [5]. Hematologically, megaloblastic anemia remains the most common presentation, characterized by macrocytosis, ovalocytes, anisocytosis, and the pathognomonic hypersegmented neutrophils on peripheral blood smear. Severe B12 deficiency can cause Ineffective intramedullary erythropoiesis, resulting in elevated LDH, indirect hyperbilirubinemia, pancytopenia, and schistocytes, which, in combination with thrombocytopenia, can closely mimic a TMA [6]. Additional manifestations include atrophic glossitis and angular cheilitis. Notably, folate supplementation without concurrent B12 repletion risks masking hematological changes due to the methylfolate trap while neurological deterioration progresses silently [6].

In our patient, serum B12 of 163 pg/mL was slightly lower than normal levels (211-911 pg/mL). However, serum B12 reflects circulating protein-bound cobalamin rather than intracellular bioavailability and can be low-normal even when tissue-level stores are more critically depleted. The markedly elevated MMA of 985 nmol/L, a metabolite that accumulates when B12-dependent methylmalonyl-CoA mutase activity is impaired, confirms significant functional cobalamin deficiency, underscoring the importance of reflex MMA testing when cobalamin deficiency is suspected clinically, as serum B12 alone may underestimate the true degree of metabolic insufficiency [6].

In B12 deficiency, the functional deficiency of THF impairs thymidylate synthesis, a rate-limiting step in DNA synthesis, due to which DNA replication is slowed while RNA synthesis and cytoplasmic development are relatively unaffected, producing large, immature cells with an open, lacy nuclear chromatin pattern and a nucleus-to-cytoplasm maturation dissociation across all three hematopoietic cell lines. In the erythroid lineage, megaloblastic erythroid precursors undergo increased intramedullary destruction through apoptosis, a process termed ineffective erythropoiesis, leading to premature death of precursors within the marrow, releasing hemoglobin breakdown products, leading to reduced haptoglobin, elevated unconjugated bilirubin, LDH, and elevated serum iron with high transferrin saturation mirroring the profile of hemolysis [15]. The reticulocyte count is characteristically and inappropriately low despite severe anemia, reflecting intramedullary destruction of megaloblastic erythroid precursors rather than peripheral hemolysis, in contrast to expected compensatory reticulocytosis in hemolytic anemia. Peripheral smear shows macrocytic cells, anisopoikilocytosis, and schistocytes due to fragmentation of structurally abnormal erythrocytes during their passage through the microcirculation in the bone marrow, a mechanism distinct from endothelial injury-driven microthrombi of true TMA [10]. In the myeloid and megakaryocytic lineage, giant metamyelocytes, hypersegmented neutrophils, and hypolobulated megakaryocytes with reduced platelet production are characteristic. If B12 levels are normal, elevated MMA and total homocysteine are the most sensitive functional biomarkers of B12 deficiency, with MMA being uniquely specific to B12 deficiency, as homocysteine elevation occurs in both B12 and folate deficiency [6]. The combination of thrombocytopenia, macrocytosis, schistocytes, elevated LDH, low reticulocyte production index, indirect hyperbilirubinemia, and response to B12 treatment, arising from this intramedullary process in the absence of true endothelial injury or ADAMTS13 deficiency, explains the biologic plausibility of TMA/pseudo-TTP in the setting of severe B12 deficiency [12].

Platelet transfusion in TTP is administered with caution, given the associated risk of arterial thrombosis and increased mortality from exogenous platelet consumption by ultra-large VWF multimers, which can propagate microvascular thrombosis. In our patient, transfusion was necessary in the setting of refractory thrombocytopenia despite plasmapheresis and high bleeding risk that outweighed thrombotic concerns. While an altered mental status was noted on presentation, this was attributed primarily to sepsis-associated encephalopathy, given the patient's early clinical recovery with antimicrobial therapy, rather than a direct neurologic manifestation of B12 deficiency or cerebral microthrombi as seen in true TTP. Similarly, the acute kidney injury is likely related to sepsis and the metabolic consequences of severe anemia rather than thrombotic microvascular occlusion of the renal vasculature.

Accurate differentiation between true TTP and pseudo-TTP has significant therapeutic implications: True TTP mandates urgent PLEX and immunosuppression, whereas pseudo-TTP due to vitamin B12 deficiency responds to cobalamin replacement alone and does not require PLEX, which can be potentially harmful due to procedural risks, complications, and a considerable resource burden. Failure to recognize this distinction may expose patients to avoidable iatrogenic harm while delaying curative therapy.

## Conclusions

In conclusion, pseudo-TMA in the setting of severe vitamin B12 deficiency is a diagnostically challenging but treatable mimic of TTP, where delayed recognition carries a significant risk of iatrogenic harm. The presence of marked macrocytosis, disproportionately elevated lactate dehydrogenase, and an inadequate reticulocyte response should prompt early consideration of nutritional deficiency as an underlying etiology, thereby avoiding unnecessary PLEX, immunosuppression, and intensive care-level interventions. Timely initiation of vitamin B12 replacement, once recognized, is both curative and cost-effective, underscoring the broader imperative for clinician awareness of this entity across hematology, critical care, and general medicine practices.
